# Health-related quality of life and its determinants among patients with psoriasis at a referral hospital in Northwest Ethiopia

**DOI:** 10.3389/fmed.2023.1183685

**Published:** 2023-07-13

**Authors:** Eyayaw Ashete Belachew, Gashaw Sisay Chanie, Eshetie Gizachew, Ashenafi Kibret Sendekie

**Affiliations:** ^1^Department of Clinical Pharmacy, School of Pharmacy, University of Gondar, Gondar, Ethiopia; ^2^Department of Information System, College of Informatics, University of Gondar, Gondar, Ethiopia

**Keywords:** psoriasis, health-related quality of life, determinants, Dermatology Life Quality Index, Ethiopia

## Abstract

**Objectives:**

This study assessed health-related quality of life (HRQoL) and its determinants among patients with psoriasis having follow-ups at the University of Gondar Comprehensive Specialized Hospital (UoGCSH).

**Design, setting, and participants:**

A cross-sectional institutional-based study was conducted at the dermatologic clinic of UoGCSH from June to August 2022. Four hundred eligible patients with psoriasis were included in the study using a systematic random sampling technique.

**The main outcome measured:**

The Dermatology Life Quality Index (DLQI) measurement scale was used to assess HRQoL. The relationship between HRQoL and independent predictor variables was investigated using bivariable and multivariate logistic regression analyses. Statistical significance was defined as a *p*-value of <0.05 at the 95% CI.

**Results:**

Of the 422 study subjects approached, 400 respondents with psoriasis were included in the final analysis. The mean (±SD) age was 39.8 (±17.2) years, and 56% were women. The most commonly prescribed medications were topical corticosteroids (68.3 %). The mean (±SD) DLQI was 13.05 (7.82). More than three-fourths (78.5%) of the patients' HRQOL was affected, and its severity ranged from very large to extremely large. Being male (adjusted odd ratio) (AOR) = 0.373, 95% CI (0.171, 0.773), the use of alternative therapy (AOR) = 0.237, 95% CI (0.114, 0.494), duration of diseases (AOR) = 0.184, 95% CI (0.061, 0.557), duration on medication (AOR) =3.75, 95% CI (1.32, 10.73), presence of comorbidity (AOR) = 6.199, 95% CI (1.921, 20.00), and income were found to have a significant association with poor HRQoL.

**Conclusion:**

Psoriasis patients had reduced HRQoL, which was lower than that of the normal population. The study identified that several variables contributed to this reduced HRQoL. Predictors that take into account interventions were essential for preserving patients' HRQoL.

## Introduction

Psoriasis is an immune-mediated skin disorder that has persistent inflammatory effects on the skin, joints, or both (1, 2). The World Health Organization (WHO) recognized psoriasis as a severe, non-transmittable skin condition and underlined the need for a proper understanding of the illness's consequences and burden on society as a whole. It affects at least 100 million individuals worldwide, with a prevalence rate ranging from 0.09 to 11.43% (3). In Ethiopia, psoriasis affects 5.4% of the population, according to a survey (4).

Systemic inflammation may increase the risk of a number of medical conditions because it has been associated with insulin resistance, type II diabetes, obesity, and cardiac events in psoriasis (5). In comparison to other systemic chronic diseases that harm internal health, patients with psoriasis have interpersonal feelings, disordered appearance, social issues, and a generally lower quality of life (QOL) (6). Studies show that psoriasis is linked to higher rates of depression, anxiety, wrath, and shame, which causes social withdrawal and absences from work and school. Significant economic and financial ramifications follow (7). The WHO recommends multilateral psoriasis management that looks beyond the ailment's physical manifestations to pinpoint the causes of low QOL (3).

Although there is no known cure for psoriasis, there are numerous efficient treatment options. With the help of new medications, symptoms can be significantly reduced, and QOL can be improved (8). Topical pharmaceuticals, light therapy, systemic anti-inflammatory drugs, and, more recently, biological agents are some of the available treatments (9). According to surveys, patients with moderate to severe psoriasis are undertreated in many countries, leaving a significant portion without good disease control. Additionally, a large portion of patients are unhappy with their care (10). As there is no universally agreed definition of what constitutes a successful therapy, the provision of efficient, practical, and secure pharmaceuticals should be part of patient care, as should the consideration of patient-reported outcomes such as treatment preferences, satisfaction, and QOL (11). QOL is an essential measure since it determines treatment goals, prognosis, and disease management (12).

There is an astonishing dearth of data on the prevalence, treatment strategy, and HRQoL associated with the most common skin illnesses, including psoriasis, at the community level in Africa. The expense of managing psoriasis is often significant due to long-term drug use and increasing prices from sophisticated therapy; this is predicted to be much more so in developing countries. According to studies, there is a substantial disparity between the effective psoriasis medicines available and the psoriasis management scenario in Africa (13).

Even though psoriasis is the most prevalent and severe form of skin disease, there is a lack of information in Ethiopia, and efforts must be made to enhance overall psoriasis management. Hence, there has been no one study conducted in the study area to determine the rate and determinants of HRQoL. As a result, the necessity for more research in this field is undeniable. Knowing HRQoL and its determinants is one of the critical steps in improving patient QOL. At the national level, the results of the current study may contribute to showing the magnitude of the problem and predictors of decreased HRQoL. Additionally, identifying the factors that impact patients' HRQoL is essential to facilitate the development of holistic approaches for relieving the patient burden. Furthermore, the results of the study can also be used by researchers who are interested in further studies in the area and by policymakers and other organizations that have a responsibility for improving HRQoL. Therefore, this study assessed HRQoL and related factors among patients with psoriasis at the UoGCSH.

## Methodology

### Study design and settings

A facility-based cross-sectional survey through patient interviews and retrospective chart review was used among patients with psoriasis at an ambulatory dermatologic clinic at UoGCSH from June to August 2022.

### Study population and sampling

Patients with the diagnosis of psoriasis aged 18 years and above who attended their follow-up at UoGCSH during the study period were included. The research participants must have undergone treatment over the previous 6 months to be included. Individuals who were severely ill and unable to speak, patients with two or more questions unanswered in their DLQI questionnaires, and patients with incomplete medical records were excluded from the study.

The sample size was calculated using a single population proportion formula, and 422 people were included. P (proportion distribution) was taken at 0.5 (50%) since no prior study had been performed on the HRQOL of psoriasis patients; 5% absolute precision, or margin of error; 5% significance; 95% confidence level; and 10% contingency were used for potential non-response and/or incomplete records.

To approach the study samples, a systematic sampling procedure was used. According to the UoGCSH Statistics and Information Office's Annual Report on Health Services and Employees, 884 psoriasis patients received follow-up care in the dermatology clinic at UoGCSH in the previous 3 months. As a result, 884/422 = 2 is the sample fraction (*k*-interval). The initial study subject was randomly chosen by the investigator, and then every other participant was chosen until the final sample size was achieved. Their related medical records were then collected, and pertinent information was gathered and discussed with the chosen respondent.

### Data collection tools and procedures

The questionnaire was prepared by reviewing the previously published literature related to the study objectives. Patients were interviewed and their medical records were examined to gather information using a structured data abstraction tool. The tool's initial section included socio-demographic and behavioral data (age, sex, educational status, monthly income, occupation, alcohol intake, khat chewing, and smoking status). A tool to gather pertinent clinical information, such as the type of psoriasis, age at initial diagnosis, duration of the disease, and comorbidities, was included in the second portion. The final section was the DLQI questionnaire. It is designed to improve the HRQoL of adults with skin diseases. It consisted of 10 questions about the patients' perception of how skin conditions have affected various elements of their lives over the previous week. It has been translated into more than 110 languages, including Amharic (the local language used to interview participants in this study), and is frequently used in clinical trials across more than 80 nations. The DLQI is a 10-item questionnaire that asks about daily activities, leisure time, employment or school, personal relationships, symptoms and emotions, and treatment (14).

### Data quality control

The study questionnaire was carefully adapted to collect all the necessary information. Before collecting data, data collectors received training. Before gathering actual data, a pretest was conducted to ensure that the data collection tool was uniform and easily understandable. The pretest was done by systematically selecting people's medication records. If necessary, improvements will be made to the data collection tool. The analysis excluded pre-tested patient information. Trained nurses collected the data, and the investigator followed closely. In addition, the checklists were rechecked for any missed, incorrect, or unreadable information while collecting data.

### Data analysis and management

After being coded and cleaned using Epi-Data version 3.1 software, the acquired data were input and analyzed using the statistical package for social sciences (SPSS) version 26 software. SPSS analysis was used to perform all statistical tests. The study of patient characteristics, including frequency, means, standard deviations (SDs), and percentiles, was conducted using summary statistics.

Continuous data were reported as mean SD and percentages, while categorical data were reported as percentages and mean standard deviation. The DLQI scores were divided into two categories: >5 and ≤5 to represent severely affected and less severely affected QOL, respectively (15). Logistic regression analysis was used to investigate the relationship between independent variables and HRQoL. An odds ratio (OR) with a 95% confidence interval (CI) was calculated to assess the degree of correlation between the predictor and outcome variables. After conducting a bivariable analysis of each variable's relationship to the dependent variable, variables with *p*-values <0.25 were selected to be included in the multivariable regression. If the probability value is <0.05, it is considered statistically significant. Tables and figures are used appropriately to depict the data processing results.

### Ethical considerations and confidentiality

The Institutional Review Board (IRB) of the University of Gondar provided ethical clearance and approval (SOP/257/2022). To obtain their acceptance and cooperation, the study goal was explained to the department heads of dermatology and hospital directors. Written informed consent was required from the patients. By eliminating patient identification and providing a code number, patient confidentiality was maintained. Participants in the study had the option to decline participation.

### Operational definitions

Initially diagnosed at age (16).

- Early onset: diagnosed before age 40 years.
- Late onset: diagnosed at or after the age of 40.

✓ Alternative medicine: The terms “complementary medicine” or “alternative medicine” refers to a broad set of healthcare practices that are not part of that country's own tradition or conventional medicine and are not fully integrated into the dominant healthcare system. They are used interchangeably in traditional medicine in some countries (17).
✓ Habitual drinking or khat chewing was defined as at least 1 drink or khat chew per week (14).
✓ Monthly income (Ethiopian birr) is grouped as very low (≤860), low (861–1,500), average (1,501–3,000), above average (3,001–5,000), and high (≥5,001) (16).
✓ Incomplete medical record: medical records with missing data from the questionnaire.
✓ The DLQI tool states that the following criteria must be fulfilled (18).

The interpretation of the DLQI tool (18).

✓ **No effect:** was defined as a DLQI score of 0–1.
✓ **The small effect:** DLQI score of 2–5.
✓ **Moderate effect:** DLQI scores of 6 and 10.
✓ **Very large effect**: The DLQI score was more than 11–20.
✓ **Extremely large effect**: The DLQI score was 21–30.

### Patient and public involvement

No patients or members of the public were engaged in the planning, execution, or interpretation of this study, and neither group will be involved in the release of the findings.

## Results

### Sociodemographic information on study participants

Of the 400 participants included in the final analysis, the mean (±SD) age of the study subjects was 39.8 (±17.2) years. More than half (56%) were female subjects, and 55.2% were married. More than half of the participants were urban dwellers (60%). Most patients (99%) had no medication availability problem, and 375 (93.2%) participants reported having a medication affordability issue. Approximately half (48.3%) of the participants used alternative medicines in addition to the treatment offered by the hospitals. The majority did not have the habit of smoking (339, 84.7%) or khat chewing (372, 93%) (Table 1).

**Table 1:** Sociodemographic characteristics of patients with psoriasis at the dermatologic clinic of UoGCSH, Ethiopia 2022.

**Variables**	**Category**	***N* (%)**
Sex	Male	176 (44)
	Female	224 (56)
Age of participants	Mean (±SD)	39.79 (17.17)
	18–35	211 (52.8)
	36–60	130 (32.5)
>60	59 (14.8)
Residence	Urban	240 (60)
	Rural	160 (40)
Marital status	Single	124 (31)
	Married	221 (55.2)
	Divorced	15 (3.8)
	Widow	40 (10)
Educational status	No formal education	89 (22.3)
	Primary (1–8)	93 (23.3)
	Secondary (9–12)	107 (26.8)
	College and above	111 (27.8)
Occupation	Farmer	90 (22.5)
	Government employee	71 (17.8)
	Business/self-employee	87 (21.8)
	Student	72 (18)
	Homemaker	71 (17.8)
	Others	10 (2.5)
Monthly income	≤ 860	147 (36.8)
	861–1,500	77 (19.3)
	1,501–3,000	82 (20.5)
	3,001–4,999	67 (16.8)
	≥5,000	27 (6.8)
Health insurance	Yes	233 (58.3)
	No	167 (41.7)
Alcohol drinking	No	167 (41.8)
	Yes	233 (58.2)
Khat chewing	Yes	28 (7)
	No	372 (93)
Cigarette smoking	No	339 (84.7)
	Yes	61 (15.3)
Medication availability	Yes	397 (99.2)
	No	3 (0.8)
Medication affordability	Yes	375 (93.2)
	No	25 (6.8)
Use of alternative medicine	Yes	193 (48.3)
	No	207 (51.7)

### Clinical characteristics of patients with psoriasis disorders

Less than two-thirds (64.2%) of patients started to experience psoriasis symptoms before age 40. More than half (53%) of the participants had the disease for <5 years. The most commonly identified form of psoriasis was plaque psoriasis (33.3%). Approximately 34% of the patients were living with at least one type of co-existing disease, including cardiovascular (10.5%), respiratory (10%), and endocrine (3.75%). The majority of the participants used topical modes of treatment (273, 68.3%), and their HRQoL was largely affected (314, 78.5%) (Table 2).

**Table 2:** Clinical characteristics of patients with psoriasis at an ambulatory dermatologic clinic of UoGCSH, Ethiopia 2022.

**Variable**		***N* (%)**
Age at initial diagnosis	< 40 years	257 (64.2)
	≥0 years	143 (35.8)
Duration of disease	< 5 years	255 (63.7)
	≥5 years	147 (36.3)
Duration of medication	< 3 years	258 (64.5)
	≥3 year	141 (35.5)
Type of psoriasis	Plaque psoriasis	133 (33.3)
	Sebopsoriasis	70 (17.5)
	Scalp psoriasis	98 (24.5)
	Palmoplantar psoriasis	44 (11)
	Pustular psoriasis	55 (13.8)
Comorbidity	Yes	136 (34)
	No	264 (66)
Common co-existing chronic diseases	Respiratory diseases	40 (10)
	Cardiovascular diseases	42 (10.5)
	Endocrine disorders	15 (3.75)
	Urinary diseases	13 (3.25)
	Musculoskeletal disorders	10 (2.5)
	Other	16 (4.0)
Treatment modality	Topical	273 (68.3)
	Systemics	88 (22)
	Both topical and systemic	39 (9.8)
Health-related QOL	Small affected	86 (21.5)
	Largely affected	314 (78.5)

### The severity of psoriasis

**Figure 1:**
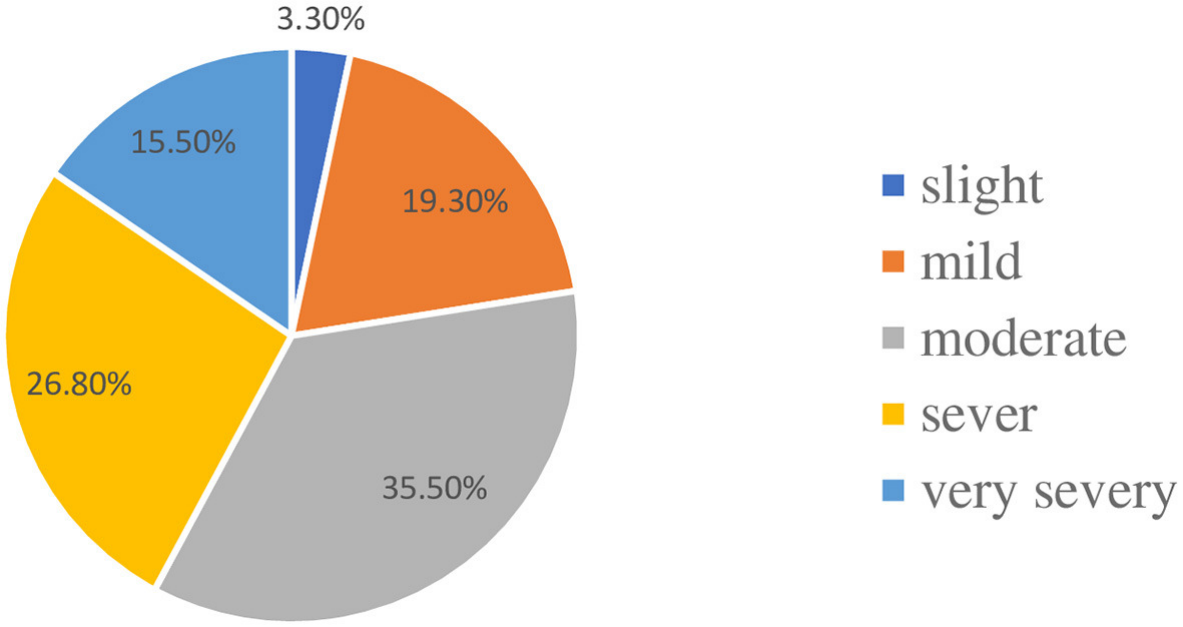
Distribution of the participants with respect to the severity of psoriasis.

In terms of psoriasis severity, a higher proportion of participants [more than three-fourths (35.5%)] had moderate psoriatic diseases, while only 3.3% had slight psoriatic diseases, according to physician diagnosis (Figure 1).

### Prescribing pattern of anti-psoriatic medications in dermatologic

The majority of the individuals in this study were being treated for psoriasis with topical corticosteroids. The most commonly prescribed steroids are betamethasone dipropionate, betamethasone dipropionate plus amphotericin B, and mometasone furoate at 62 (15.5%), 41 (10.3%), and 86 (21.5%), respectively. For plaque psoriasis, mometasone furoate 31 (23.3%) is the most frequently prescribed topical corticosteroid. While for scalp psoriasis, betamethasone dipropionate (17.3%) is more often used, and other topical agents such as tretinoin cream (71, 17.8%) were mostly used for plaque psoriasis (28, 21.1%). Methotrexate was used by only 18 (4.5%) participants (Table 3).

**Table 3:** Prescribing pattern of anti-psoriatic medications dermatologic clinic of UoGCSH, Ethiopia 2022.

**Treatments**	**The type of psoriasis**					**Total *N* (%)**
	**Plaque psoriasis** ***N*** = **133**	**Sebo psoriasis** ***N*** = **70**	**Scalp psoriasis** ***N*** = **94**	**Palmoplantar psoriasis** ***N*** = **44**	**Pustular psoriasis** ***N*** = **55**	
**Topical corticosteroids**						
Betamethasone dipropionate	23 (17.3)	15 (21.4)	17 (17.3)	6 (10.6)	1 (1.8)	62 (15.5)
Betamethasone dipropionate plus amphotericin B	8 (6)	4 (5.7)	5 (5.1)	1 (2.3)	23 (41.8)	41 (10.3)
Betamethasone dipropionate plus ketoconazole	8 (6)	7 (10)	1 (1)	3 (6.8)	1 (1.8)	20 (5)
Betamethasone valerate	3 (2.3)	5 (7.1)	6 (6.1)	4 (9.1)	3 (5.5)	21 (5.3)
Clocortolone pivalate	5 (3.8)	10 (14.3)	8 (8.2)	7 (15.9)	5 (9.1)	35 (8.3)
Mometasone furoate	31 (23.3)	15 (21.4)	13 (13.3)	12 (27.3)	15 (27.3)	86 (21.5)
Clobetasone propionates	9 (6.8)	6 (8.6)	4 (4.1)	5 (11.4)	2 (3.6)	26 (6.5)
**Other topical agents**						
Salicylic acid	3 (2.3)	0 (0.0)	2 (2)	1 (2.3)	2 (3.6)	8 (2)
Fusidic acid	12 (9)	0 (0.0)	0 (0.0)	0 (0.0)	0 (0.0)	12 (3)
Tretinoin cream	28 (21.1)	1 (1.4)	40 (40.8)	0 (0.0)	2 (3.6)	71 (17.8)
**Systemic agent**						
Methotrexate	3 (2.3)	7 (10)	2 (2)	5 (11.4)	1 (1.8)	18 (4.5)
Total						400 (100%)

### Health-related quality of life of the participants

**Figure 2:**
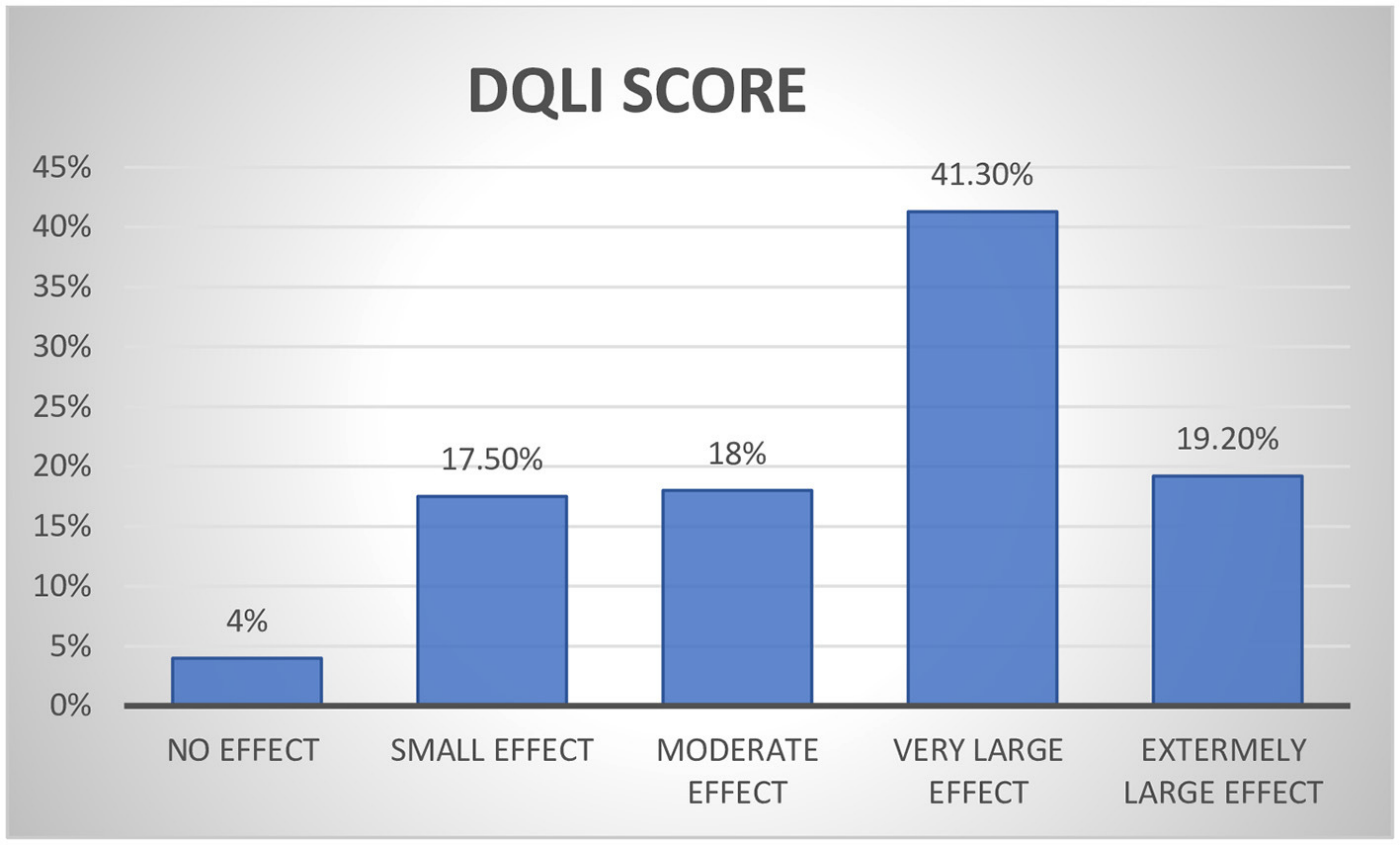
Proportion of overall effect of psoriatic on the quality of life among psoriasis patients.

According to the DLQI, 19.2% of patients had an extremely big impact on their HRQoL, whereas 17.5% of patients reported a small impact (Figure 2).

### Respondents' quality of life domains

The results revealed that the affected domains of psoriatic patients varied. The score for each domain showed that the scores for work and school were lower at 1.58 (±0.70), and the treatment score was higher at 4.05 (±0.94), which was found to be the most affected domain. The overall mean DLQI was found to be 13.05 (±7.82), corresponding to a very large effect (Table 4).

**Table 4:** Mean score for each domain of the participant dermatologic clinic of UOGCSH, Ethiopia 2022.

**Domain**	**Mean (±SD)**
Symptoms and feelings	2.08 (0.98)
Daily activities	3.34 (1.14)
Leisure	3.73 (1.04)
Work and school	1.58 (0.70)
Personal relationships	3.15 (1.01)
Treatment	4.05 (0.94)
Overall DLQI	13.05 (7.82)

### Factors associated with poor quality of life among participants

To identify the determinant of poor HRQoL among patients with psoriasis, a bivariable analysis was performed. Accordingly, age, sex, residence, educational status, income, healthcare service, alcohol use history, khat chewing, duration of medication, duration of diseases, traditional medicine, and treatment modality were considered for multivariate analysis (*p* < 0.25). In multivariate analysis, sex, monthly income, use of traditional medicine, duration of disease, duration of medication, and comorbidity were strongly correlated with poor HRQoL. The odds of having poor HRQoL for male patients were reduced by 67% [AOR = 0.373 (95% CI: 0.171, 0.773); *P* = 0.009]. This study also found a significant association between monthly income and poor HRQoL at incomes of <860 [AOR = 0.073 (95% CI: 0.007, 0.73); *P* = 0.026], at incomes of 861–1,500 [AOR = 0.076 (95% CI: 0.007, 0.789); *P* = 0.031], at incomes of 150–3,000 [AOR = 0.031 (95% CI: 0.003, 0.298); *P* = 0.002], and at income of 300–4,999 [AOR = 0.045 (95% CI: 0.005, 0.411); *P* = 0.008]. There was also a significant association between the use of traditional medicine, duration of medication, and comorbidities [AOR = 0.237 (95% CI: 0.114, 0.494); *P* < 0.001], [AOR = 3.75 (95% CI: 1.32, 10.73); *P* = 0.013], and [AOR = 6.199 (95% CI: 1.921, 20.00); *P* = 0.002], respectively (Table 5).

**Table 5:** Factors associated with poor quality of life among patients with psoriasis attending the dermatologic clinic of UOGCSH, Ethiopia 2022.

**Variables**	**DLQI**		**COR (95%CI)**	***P*-value**	**AOR (95%CI)**	***P*-value**
	≤ **5 (*****N*** = **86)**, >**5 (*****N*** = **314)**					
**The age category**						
18–35	63 (29.9)	148 (70.1)	0.082 (0.020, 0.348)	0.001	0.58 (0.100, 3.38)	0.54
36–60	21 (16.2)	109 (83.8)	0.182 (0.041, 0.804)	0.025	0.75 (0.132, 4.23)	0.74
≥ 60	2 (3.4)	57 (96.6)	1		1	
**Sex**						
Male	40 (22.7)	136 (77.3)	0.879 (0.54, 1.14)	0.593	0.373 (0.171, 0.773)	**0.009**
Female	46 (20.5)	178 (79.5)	1		1	
**Residence**						
Urban	73 (30.4)	167 (69.6)	0.202 (0.108, 0.380)	**< 0.001**	0.405 (0.202, 1.204)	0.541
Rural	13 (34.4)	147 (65.6)	1		1	
**Educational status**						
No formal education	6 (6.7)	83 (93.3)	11.75 (4.73, 29.17)	< 0.001	2.02 (0.47, 8.57)	0.337
Primary (1–8)	10 (10.8)	83 (89.2)	7.05 (3.31, 15.00)	**< 0.001**	2.64 (0.88, 8.11)	0.081
Secondary (9–12)	19 (17.8)	88 (82.2)	3.93 (2.11, 7.32)	< 0.001	2.48 (1.01, 6.08)	0.047
College and above	51 (45.9)	60 (54.1)	1		1	
**Monthly income**						
< 860	19 (12.9)	128 (87.1)	0.25 (0.033, 2.02)	**0.19**	0.073 (0.007, 0.73)	**0.026**
861–1,500	9 (11.7)	68 (88.3)	0.291 (0.035, 2.40)	**0.25**	0.076 (0.007, 0.789)	**0.031**
1,501–3,000	31 (37.8)	51 (62.2)	0.063 (0.008, 0.49)	**0.008**	0.031 (0.003, 0.298)	**0.002**
3,001–4,999	26 (38.8)	41 (61.2)	0.061 (0.008, 0.471)	**0.008**	0.045 (0.005, 0.411)	**0.008**
>5,000	1 (3.7)	26 (96.3)	1		1	
**Health insurance**						
No	30 (12.9)	203 (87.1)	3.41 (2.07, 5.67)	**< 0.001**	1.402 (0.68, 2.68)	0.354
Yes	56 (33.5)	111 (66.5)	1		1	
**Alcohol uses history**						
No	52 (31.1)	115 (68.9)	2.64 (1.62, 4.31)	**< 0.001**	0.550 (0.253, 1.170)	0.120
Yes	34 (14.6)	199 (85.4)	1		1	
**Khat chewing**						
Habitual	3 (10.7)	25 (89.3)	2.39 (0.70, 8.12)	0.16	2.42 (0.498, 11.789)	0.273
Not habitual	83 (22.3)	289 (77.7)			1	
**Use of traditional medicine**						
Yes	68 (35.2)	125 (64.5)	0.175 (0.099, 0.309)	< 0.001	0.237 (0.114, 0.494)	**< 0.001**
No	18 (8.7)	189 (91.3)	1		1	
**The duration of the disease**						
< 5 years	64 (25.1)	151 (74.9)	0.53 (0.31, 0.91)	0.021	0.184 (0.061, 0.557)	**0.003**
≥5 years	22 (15.2)	123 (84.8)	1		1	
**Duration of medication**						
< 3 years	58 (22.5)	200 (77.5)	0.85 (0.51, 1.41)	0.24	3.75 (1.32, 10.73)	**0.013**
≥3 years	28 (19.9)	113 (80.1)	1			
**Comorbidity**						
Yes	6 (29.2)	130 (70.8)	9.42 (3.98, 22.25)	< 0.001	6.199 (1.921, 20.00)	**0.002**
No	80 (30.3)	184 (69.7)	1			
**Treatment modality**						
Topical	60 (22)	213 (78)	0.91 (0.40, 2.09)	0.083	1.16 (0.391, 3.483)	0.785
Systemics	18 (20.5)	70 (79.5)	1.04 (0.39, 2.55)	0.90	0.600 (0.171, 2.907)	0.423
Both	8 (20.5)	31 (79.5)	1		1	

Bold figures at *p*-value show variables at a *p*-value of < 0.05.

## Discussion

To evaluate the effectiveness of medical and healthcare interventions, it is vital to measure patients' perceptions of their HRQoL (19). In this institutional-based study, the DLQI tool was applied to the HRQoL of patients with psoriasis. This study's findings showed that the overall mean DLQI score was 13.05, which corresponds to numerous effects on patients' HRQoL.

This result is equivalent to that of earlier Chilean investigations (20) and Egypt (21), which reported mean DLQI scores of 14 and 12.2, respectively. In contrast, this outcome was higher than that of a study conducted in Ethiopia (16), with a mean score of 6.25, and in Turkey (22), with a mean score of 7.03. Even though the study was conducted in referral hospitals with better facilities and expertise, the patients' QOL was highly compromised. This might be due to a difference in sample size since this study enrolled a higher number of participants, which may result in such deviations in observations.

From the approached respondents, 41.3 and 19.2% of patients, respectively, replied that the disease has a countless and extremely endless impact on their QOL. This outcome resembles studies from India (23) and Malaysia (24), which discovered frequent to extremely significant effect estimates of 31.7 and 30%, respectively, in both countries. However, a study from Alert Hospital in Ethiopia (16) found that only 13% of participants had a significant impact from psoriasis, which is lower than the outcome of this study. However, studies from Egypt (21) and South Africa (25) found higher results of 60 and 50.7%, respectively. These variations might be explained by variations in the capabilities of the medical centers and the medical staff in terms of the healthcare services they offered. In the case of an alert hospital, for instance, the hospital is a dermatology specialist hospital in the nation and may offer patients superior healthcare than other facilities. Patients may be able to maintain a higher degree of HRQoL as a result of this.

The current study also revealed that “treatment” was the area of HRQoL impairment caused by psoriasis that was most severely impacted, with a total value of mean 4.05, while “leisure” was the second most affected domain, with a total value of mean 3.73. The impact of psoriasis was less in “symptoms and feelings” (mean 2.08), “personal relationships” (3.15), and “work and school” (1.58). This result is contrary to studies from India (26) and South Africa (27), which have reported that “daily activities” are the most affected domains. On the other hand, studies from Thailand (28) and Malaysia (24) found symptoms and feelings as the highest domain and daily activities as the second. These differences might result from the participants' sociocultural differences and differences in their level of knowledge and attitudes toward their dermatological conditions, which may affect their self-care and management practices.

The independent predictors of poor QOL in this study included gender, monthly income, duration of disease, duration of medication, and comorbid diseases. Thus, patients with psoriasis frequently struggle socially and psychologically because of their surroundings, have issues with their self-image and self-esteem, feel stigmatized, and feel ashamed and embarrassed about their appearance (8). This study showed that poor HRQoL was highly associated with the duration of the disease (*p* = 0.001); the longer the duration of the disease, the less likely it is that patients will have a worse QOL. Similarly, a study from Spain (29) found a similar association. The reason could be that psoriasis is a chronic disease, and over the years, people become used to living with it, or perhaps patients get used to having the illness in their lives. Contrary to this, studies from Germany (30) and Poland (31) found that the long duration of the disease worsens HRQoL. The discrepancy could be due to differences in personality traits and Western and sociocultural differences. However, a study from China (14) did not observe a significant association between disease duration and poor HRQoL. This could be due to variations in variables that are included in regression analysis and different dichotomization techniques. In addition, different studies have chosen various confounding factors for regression adjustment, and cross-cultural differences in response to an HRQoL instrument specifically for dermatology have been documented.

The current study discovered that patients with psoriasis with comorbid diseases are 6.19 times more likely to have a poor QOL than those with only psoriasis. This result is congruent with research from Spain discovered that a strong correlation and a significant association between QOL and comorbidity were observed (32). This could be due to having more diseases that increase the burden of psoriasis in different aspects, including psychological and economical aspects. On the other hand, studies from Turkey (22), Chile (20), and Malaysia (24) did not observe a significant association between comorbidity and a worse quality of life. This discrepancy may be due to distinctions in variables during analysis.

Social drug habits, including alcohol drinking, cigarette smoking, and khat chewing, were not associated with worse QOL in this study. This finding agrees with studies from China (14) and India (26), which found a non-significant association. Contrarily, a study from the United Kingdom reported a significant association among alcohol consumption, cigarette smoking, and HRQOL in patients with psoriasis (33). This could be due to the difficulty of measuring alcohol or cigarette intake precisely, and assessments of alcohol, khat, and cigarette use were heavily questionnaire-based and vulnerable to bias.

In this study, sex and monthly income were independent predictors of poor HRQOL. This is consistent with studies from Chile (20) and Spain (29) that have reported a significant association between gender and poor HRQOL. However, the result is a contraindication, with findings from India (26), Thailand (28), and Malaysia (24). This variation could be the result of different cultures and participant selection processes.

Topical therapy is the mainstay of treatment for psoriasis in terms of anti-psoriasis medication prescribing patterns, with topical corticosteroids and vitamin D analogs being the two most popular options (34). Patients with moderate-to-severe psoriasis require systemic therapy. Methotrexate, cyclosporine, and acitretin are the three most commonly used systemic drugs (35). According to the findings of this study, topical corticosteroids were prescribed to most psoriasis patients (68.2%), followed by systemic agents (22%), with betamethasone dipropionate being the most commonly prescribed (15.5%). This finding is consistent with previous Ethiopian research (16, 36). However, according to Indian literature (37), clobetasol propionate is the most commonly prescribed medication. This variation could be due to differences in national treatment guidelines. Furthermore, 22.8% of our participants were given impromptu preparations containing salicylic acid or coal tar. This finding is lower than in an Indian study (37) where 54.76% of patients were prescribed salicylic acid and coal tar in addition to other topical preparations. Guidelines recommend using these agents as keratolytic agents to improve steroid permeation and effectiveness (38).

In general, the current study highlighted the status of HRQoL and determinant factors in patients with psoriasis. The findings implied that patients need to be monitored, followed, and closely monitored, focusing on the independent factors contributing to poor HRQoL.

## Study strengths and limitations

This study provides a broad review of the potential relationships between demographic factors, clinical parameters, treatment-related factors, and HRQoL in patients with psoriasis. Although there is a study on factors associated with poor QOL in psoriasis patients, as far as we are aware, this is the first research that provides a more comprehensive view of factors associated with poor HRQoL in psoriasis patients in Ethiopia. Additionally, this study included a large sample size, which may increase the validity of the study.

However, this study has limitations. First, as with all cross-sectional studies, it cannot provide evidence of the temporal relationship between exposure and outcome as the predictors and outcome were assessed simultaneously. Second, variables such as drinking, smoking, and khat chewing were liable to bias because study participants may not be honest about the frequency of their habits. Third, there was a lack of data on the current severity and body surface area involvement of the disease, which could affect the dependent variable.

## Conclusion

This study concluded that poor QOL was observed among the majority of patients with psoriasis. More than three-fourths of the study participants' QOL was largely affected. Being male, having a low monthly income, having a duration of disease <5 years, having comorbidities, and having a >3-year duration on medication made them extremely susceptible to poor QOL. Generally, highly poor HRQoL and several contributors to poor quality of life were identified. Therefore, this high prevalence of poor HRQoL should be given due attention, and future researchers should focus on medication adherence.

## Data availability statement

The raw data supporting the conclusions of this article will be made available by the authors, without undue reservation.

## Ethics statement

The studies involving human participants were reviewed and approved by the Institutional Review Board (IRB) of the University of Gondar provided ethical clearance and approval (SOP/257/2022). The patients/participants provided their written informed consent to participate in this study.

## Author contributions

All authors contributed to the study conceptualization, design, data collection, interpretation, and the first preparation of the publication. The final version was read and approved by all authors, who also provided the material and critical review.
